# Ionic Liquid-Catalyzed CO_2_ Conversion for Valuable Chemicals

**DOI:** 10.3390/molecules29163805

**Published:** 2024-08-11

**Authors:** Peng Wang, Rui Wang

**Affiliations:** School of Environmental Science and Engineering, Shandong University, No. 72 Seaside Road, Qingdao 266237, China

**Keywords:** CO_2_, valuable chemicals, ionic liquids, catalysis

## Abstract

CO_2_ is not only the main gas that causes the greenhouse effect but also a resource with abundant reserves, low price, and low toxicity. It is expected to become an important “carbon source” to replace oil and natural gas in the future. The efficient and clean resource utilization of CO_2_ has shown important scientific and economic value. Making full use of abundant CO_2_ resources is in line with the development direction of green chemistry and has attracted the attention of scientists. Environmentally friendly ionic liquids show unique advantages in the capture and conversion of CO_2_ due to their non-volatilization, designable structure, and good solubility, and show broad application prospects. The purpose of this paper is to discuss the research on the use of an ionic liquid as a catalyst to promote the synthesis of various value-added chemicals in CO_2_, hoping to make full use of CO_2_ resources while avoiding the defects of the traditional synthesis route, such as the use of highly toxic raw materials, complicated operation, or harsh reaction conditions. The purpose of this paper is to provide reference for the application and development of ionic liquids in CO_2_ capture and conversion.

## 1. Introduction

Since the Industrial Revolution, the widespread use of fossil fuels has dramatically increased CO_2_ emissions. The concentration of CO_2_ in the atmosphere has increased from 280 μL/L before the Industrial Revolution to 415 μL/L in 2021 [1]. With the increasing CO_2_ content in the atmosphere, a series of ecological and environmental problems have been caused, including the greenhouse effect, glacier melting, and climate warming [2]. In addition, the excessive consumption of fossil energy has also led to the emergence of the “carbon source crisis”. The utilization of chemical resources has shown more and more important scientific and economic value and has attracted wide attention. The use of ionic liquids to convert CO_2_ into high-value-added chemical products is of great significance to alleviate the greenhouse effect and the energy crisis. However, CO_2_ is also an abundant, low-cost, non-toxic, and renewable C1 resource. Through the development of efficient catalytic technology, CO_2_ can be converted into a series of high-value-added chemical and energy products, such as dimethyl carbonate [3], propylene carbonate [4], α-alkylene cyclic carbonate, and cyclic carbonate [5,6]. CO_2_ conversion typically involves the following steps: (1) diffusion of CO_2_, (2) uptake of CO_2_ in the presence of a catalyst, (3) conversion of CO_2_ on a heterogeneous or homogeneous catalyst surface, (4) desorption of the product from the catalyst, and (5) diffusion of the product into the solution/volume phase or into the separation [7,8]. Efficient catalytic techniques can fully utilize CO_2_ as a class of carbon-based energy molecules. So far, the industrialization process of producing organic chemicals from CO_2_ has been relatively slow. According to statistics, industrial CO_2_ consumption accounts for only 0.36% of the total global emissions, indicating that there is still a huge space for development in the utilization of CO_2_ resources [9]. Due to the thermodynamic stability and kinetic inertia of CO_2_, the high efficiency and high selectivity of CO_2_ conversion require rather harsh reaction conditions. Therefore, it is particularly important to develop new technologies for CO_2_ catalytic conversion and find new ways of CO_2_ resource utilization in order to achieve high-value utilization of CO_2_ under mild conditions.

**Figure 1:**
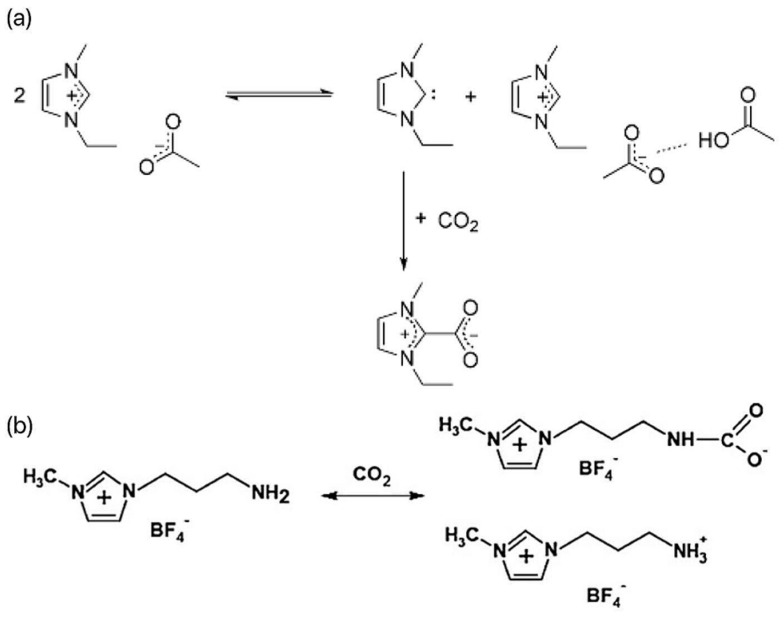
(**a**) Reaction process of CO_2_ and [EMIm]Oac [12]. (**b**) Mechanism of CO_2_ capture by NH_2_-RTIL [13].

Ionic liquids (ILs) are a large family of organic salts composed of organic cations (such as imidazole, pyridine, ammonium, phosphorus, guanidine, etc.) and anions (such as Cl^−^, Br^−^, I^−^, BF_4_^−^, PF_6_^−^, etc.) [10]. ILs have the characteristics of low volatility, non-flammability, good thermal stability, and physicochemical stability, and they are considered to be excellent CO_2_ liquid absorbers [11]. Most ILs absorb CO_2_ through physical or chemical action. The physical absorption is mainly affected by the van der Waals force, and the desorption process is relatively simple. Just reduce the pressure or increase the temperature. Chemical absorption is achieved through the establishment of covalent bonds between ILs, which makes the process of CO_2_ recovery and solvent regeneration more complicated. There are two main ways in which ILs interact with carbon dioxide. First, for conventional ILs, when carbon dioxide is passed into the reaction system containing the ILs, CO_2_ can form the complex EMIm-CO_2_ with the C2-(H) of the imidazole ring, as shown in Figure 1a [12]. In this complex, CO_2_ is partially negatively charged, and its linear structure is destroyed, thus reducing the activation energy of the reaction and making CO_2_ more easily reduced by electrons. Second, the interaction between the amino-functionalized ILs and CO_2_ mainly improves the solubility of the CO_2_ in the reaction system through -NH_2_ and generates -NHCOO^−^ intermediates, as shown in Figure 1b [13]. When the ILs containing -NH_2_ participate in the photocatalytic CO_2_ reduction reaction, the -NH_2_ at the end of the alkyl chain is more likely to interact with CO_2_ to generate -NHCOO^−^, which promotes the reaction. Dong et al. found through a molecular dynamics simulation that the complex hydrogen bond structure in ILs was the main reason for improving its CO_2_ adsorption performance [14]. Conventional and functional ILs are commonly used for CO_2_ absorption and gas separation. In conventional ILs, fluorinated anions show good absorption performance, but the absorption of CO_2_ is relatively weak. However, functional ILs contain amino, hydroxyl, and other basic functional groups, which are widely used. Functional ILs can absorb CO_2_ through chemical action and enhance the absorption effect, which is the most promising type of ILs to realize industrialization at present. As early as 2002, Bates et al. proposed an amine-functionalized task-specific IL that binds CO_2_ to a novel amino-functionalized IL through physical and chemical absorption, with an absorption ratio of 1:2, significantly increasing the solubility of CO_2_ and meeting theoretical values based on chemical-absorption mechanisms [15].

## 2. Ionic Liquids

### 2.1. Imidazole Ionic Liquids

**Figure 2:**
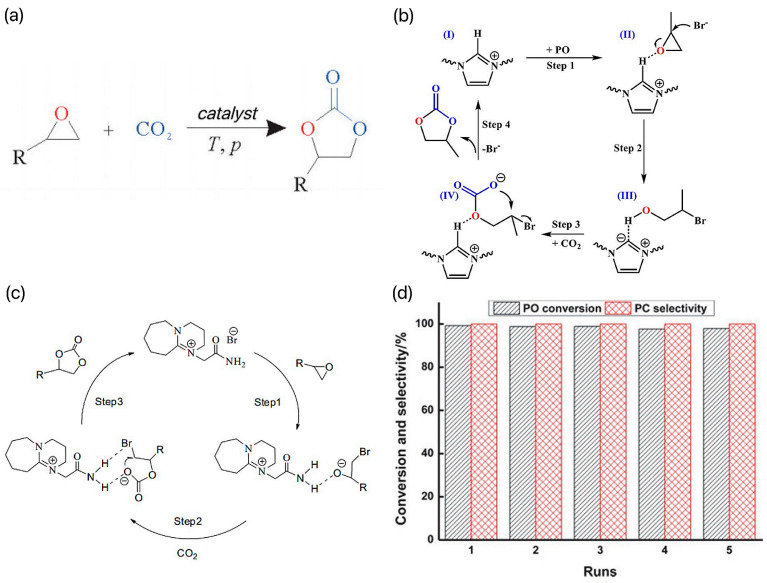
(**a**) Preparation of cyclic carbonates by the cyclization reaction of CO_2_ with small-molecule epoxides [16]. (**b**) Mechanism of CO_2_ cycloaddition over the PIL-DVBIV catalyst [18]. (**c**) Possible synthetic mechanism of propylene carbonate [19]. (**d**) The loop test results [19].

Cyclic carbonate is a class of important chemical products widely used in chemical and chemical fields, with excellent physical and chemical properties and good biodegradability. It can be used as solvents, additives, electrolytes, pharmaceutical intermediates, and polymer monomers, and is widely used in many fields [16]. At present, the preparation of cyclic carbonates by a cycloaddition reaction between CO_2_ and small molecule epoxides has become one of the research hotspots, as shown in Figure 2a. This reaction route is in line with the atom economy, and the use of CO_2_ as the carbonylation reagent replaces the use of phosgene in the traditional process, which meets the development needs of green chemistry.

At present, the reaction is catalyzed by a homogeneous KI system in industry. The reaction is efficient because there is a hydrogen bond between the oxygen in PO and the hydroxyl group in cellulose. However, the production scale of the system is small and the reaction conditions of the catalytic process are harsh (180~200 °C, 5~8 MPa) [17]. In order to reduce the energy consumption of the reaction and realize the efficient activation and conversion of CO_2_ under milder conditions, researchers developed different types of IL catalysts and investigated their catalytic performance and mechanism in the preparation of cyclic carbonate by coupling CO_2_ with epoxide. Among all the ILs, imidazolium ILs are a class of ILs containing imidazole rings in the cations because of their large number and application in many fields. Due to the unique advantages of better solubility, better electrochemical stability, wider electrochemical window, and environmental friendliness, imidazolium ILs have been used in many fields of chemistry. In recent years, the wide application of imidazolium-based ILs in CO_2_ cycloaddition reactions has attracted much attention. For example, Du et al.’s research team developed a series of directed vinyl-functionalized imidazole ILs and used these ILs for polymerization and copolymerization with 1,4-divinylbenzene (DVB) [18]. The team successfully synthesized a catalyst named PIL-DVBIV, which showed significant activity and selectivity in the CO_2_ cycloaddition reaction. Under the optimized reaction conditions (110 °C, 2.0 MPa, and 6 h), the yield of the IL-DVB-IV catalyst for propylene carbonate reached 93%, and the selectivity reached 99%. In addition, the catalyst also showed good recyclability and versatility for various epoxides, which is of great significance for industrial production. Figure 2b shows the mechanism of the CO_2_ cycloaddition reaction on the PIL-DVBIV catalyst, which is designed to efficiently convert CO_2_ to organic carbonate products. Li et al. synthesized 1,8-diazabicyclo[5.4.0]nonene (DBU), 1,5-diazabicyclo[4.3.0]-5-nonene (DBN), and imidazole (MIM)-derivatized brominated ILs, which were used to catalyze the cycloaddition reactions of CO_2_ with several epoxides to produce cyclic borates [19]. The possible synthetic mechanism of propylene carbonate is shown in Figure 2c. First, the epoxides are activated by hydrogen bond interactions, which makes the opening of the ring easier. Subsequently, the ring of the epoxide opens up on the smaller carbon atoms through a nucleophilic attack. Finally, cyclic carbonates are then formed by intramolecular cyclization nucleophilic attack. The DBU-derived bromide IL systems were found to have the best catalytic activity. The team investigated the effect of reaction conditions (temperature, pressure, and reaction time) on the reaction of CO_2_ to generate propylene oxide (PO). The results showed that the conversion of PO, and the selectivity of propylene carbonate (PC) were able to reach 99% and 99% at 120 °C, 1 MPa, and 2.5 h with 2 mol % DBU-derived bromine ILs as the catalyst. The IL system could be reused at least five times without decreasing the selectivity and conversion under optimal reaction conditions. The loop test results are shown in Figure 2d. The results of this study provide useful information for the use of imidazolium-based brominated ILs to catalyze the cycloaddition reaction of CO_2_ with epoxides and also demonstrate the feasibility and sustainability of such systems in practice.

### 2.2. Pyridine Ionic Liquids

**Figure 3:**
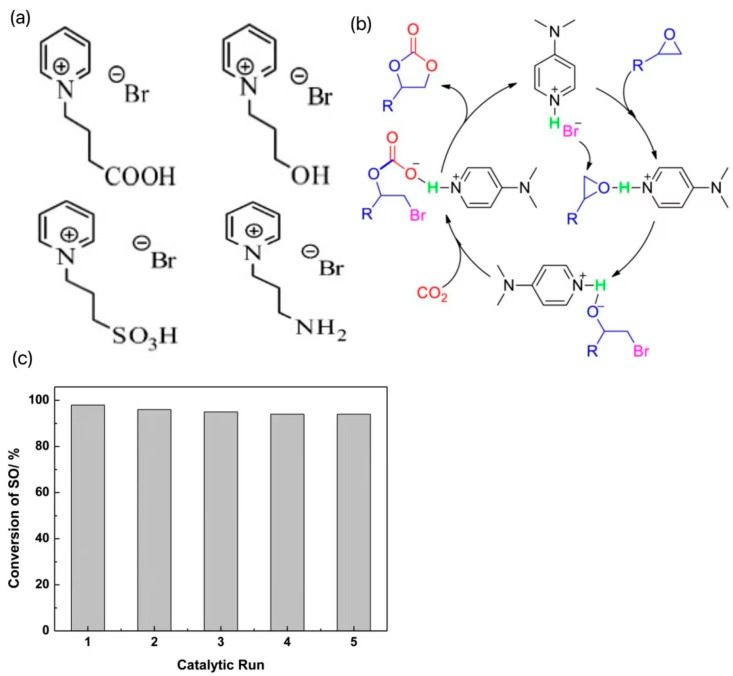
(**a**) Four new hydrogen bond donor-functionalized ILs designed after the introduction of -OH, -COOH, -NH_2,_ and -SO_3_H groups, respectively, into the structure of 1-butylpyridinium bromide ([BPy]Br) [20]. (**b**) The catalytic conversion mechanism [21]. (**c**) The cycling performance test results [21].

In contrast, pyridine ILs are less reported. Wang et al. synthesized a series of traditional pyridine ILs, introduced -OH, -COOH, -NH_2_, and -SO_3_H groups into the structure of 1-butylpyridine bromide ([BPy]Br), respectively, and designed four new hydrogen bond donor functionalized ILs as shown in Figure 3a. The effects of cationic properties, alkyl chain lengths, and anionic species on the catalytic activities were systematically studied by using theoretical calculation methods [20]. The results showed that the insertion reaction energy barrier of CO_2_ was almost negligible and did not participate in the decisive step of the reaction; the epoxide ring-opening reaction had a higher energy barrier relative to the ring-closing reaction, which showed a decisive step. Increasing the alkyl chain length can improve the catalytic activity, but the catalytic activity decreases when the alkyl chain length is increased to C4, which is attributed to the large spatial resistance of the cation that prevents the reaction from proceeding. In addition, the catalytic performances of four synthesized functionalized pyridine ILs were comparatively investigated, and the results showed that the ILs containing the carboxyl group had the highest catalytic activity for the cycloaddition reaction. The theoretical study is instructive for the further design and synthesis of functionalized pyridine ILs. Zhang et al. developed an efficient and recoverable catalyst, 4-(dimethylamino)hydrobromopyridine ([DMAPH]Br), for the generation of cyclic carbonates from atmospheric CO_2_ and epoxides, and the catalytic conversion mechanism is shown in Figure 3b [21]. In the presence of 1 mol % of [DMAPH]Br in solvent-free conditions, excellent conversion and selectivity were achieved for a variety of terminal epoxides. In addition, the catalyst could be recycled more than five times without a significant loss of catalytic activity, and the cycling performance was tested as in Figure 3c. The excellent catalytic performance of [DMAPH]Br was attributed to the synergistic effect of acidic protons and bromide ions on the epoxides and CO_2_ enhanced by the cationic positively charged off-domains. In the same period, Liu et al. developed pyridine anion-based ILs (e.g., [P_4444_][2-OP]) that could achieve efficient conversion of CO_2_ to synthesize a series of epoxides of carbonates at 30 °C and 1 MPa [22]. The high activity was attributed to the synergistic interaction of the two interaction sites in the PA-ILS anion to activate CO_2_ and the cation to activate the epoxide through coordination with the central P+ unit, which led to the high activity of the workhorse catalysts.

### 2.3. Quaternary Ammonium/Quaternary Phosphonium Salts Ionic Liquids

**Figure 4:**
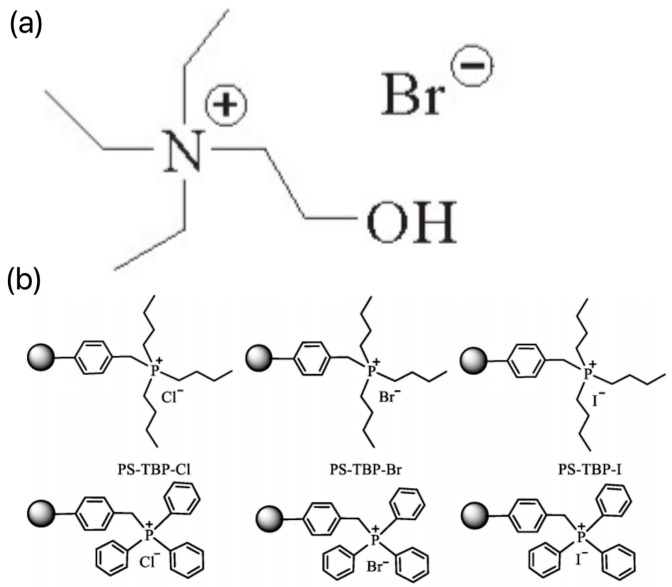
(**a**) Structure of HETEAB [23]. (**b**) A series of polymer-loaded quaternary phosphorus ILs catalysts with high grafting amount [25].

Inspired by functionalized imidazole and pyridine ions, quaternary ammonium/quaternary phosphonium ILs have attracted attention in recent years. Zhang et al. used the DFT method to theoretically investigate the mechanism of catalytic CO_2_ fixation by 2-hydroxy-ethyl-triethylammonium bromide (HETEAB), the structure of which is shown in Figure 4a [23]. The similarities and differences between the two catalysts, HETEAB and 2-hydroxy-ethyl-tributylammonium bromide (HETBAB), were further discussed. The results showed that the presence of -OH functional groups and active H atoms in ILs is an important factor in improving the performance of the catalysts. In order to better understand the reaction mechanism, LAUNAY et al. used the same hydroxyl-substituted quaternary ammonium catalysts to catalyze the coupling reaction between CO_2_ and epoxide by combining experimental and density functional theory calculations [24]. The effects of alkyl chain length and anion species on the catalytic activity were systematically investigated. Specific structural and energetic information on each step of the catalytic reaction was obtained, and the following conclusions were obtained. (1) The ring-opening process of epoxide has the highest energy barrier, which is manifested as a decisive step. (2) For the same ammonium salt cation, Br has a higher catalytic activity than C1. (3) For the same cation, the nucleophilicity of the anion is not the only factor affecting catalytic activity. It is also affected by the ion removal and is also influenced by the ion removal. It is also affected by the ion-leaving ability and spatial site resistance. Li et al. systematically designed and synthesized a series of polymer-loaded quaternary phosphorus IL catalysts with a high grafting amount, as shown in Figure 4b [25]. Based on experimental and simulation results, it was also found that the high catalytic activity of the catalyst is attributed to the weak interaction energy between anions and cations. Among them, PS TPP Cl performed well, with a low interaction energy of 72.2 kJ/mol, which led to a conversion of PO of up to 99.2% and a selectivity of PC of up to more than 99%. Even after five recoveries, its conversion of propylene oxide remained above 95% with almost no loss. Meanwhile, PS TPP Cl also shows good versatility and recyclability.

### 2.4. Loaded Ionic Liquids

Although the above-mentioned homogeneous IL catalysts have high catalytic performance, most of the ILs are difficult to recycle, so loaded ILs have been designed, i.e., the homogeneous ILs are loaded onto the carriers (e.g., carboxymethylcellulose, molecular sieve MCM-22, metal-organic framework composites, silica, etc.) [26].

**Figure 5:**
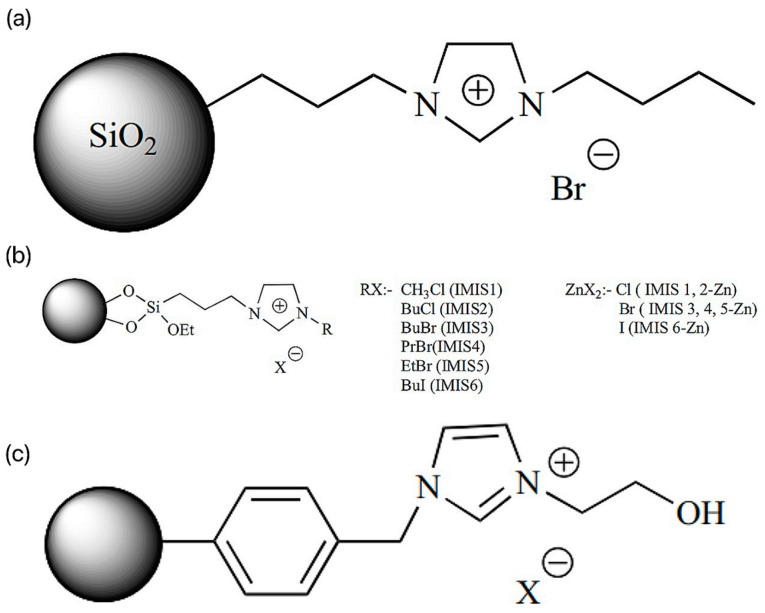
(**a**) Im-IL structure. (**b**) Structure of IMIS and IMIS-Zn. (**c**) PS-HEIMX, X = Br, Cl, I [37].

SILs are catalysts that convert carbon dioxide into various useful compounds [27]. Cyclotronic carbonates have excellent applications in different fields. Cyclotronic carbonates can be used as non-protonic solvents (polar [28,29,30], as electrolytes in lithium-ion batteries [31,32], as intermediates in reactions [33], and as monomers in polymer preparation and have practical applications in pharmaceutical sciences [34,35,36]. In this context, the researchers introduced reusable SIL Im-ILs (silica-immobilized imidazolium ILs, ILs content 1.86 mmol/g) for catalyzing the cycloaddition reaction, as shown in Figure 5a [37]. The solvent-free reaction of allyl glycerol ether (AGE) by the addition of CO_2_ at 1.76 MPa was successful in generating cyclic carbonates with the highest conversion (98.7%) (penta). Similar reactions were catalyzed by IMIS (silica-loaded various imidazolium ILs) with different halide anions and different alkyl chain lengths as in Figure 5b [37]. The silica-loaded 1-butyl-3-(3-triethoxysilylpropyl) imidazolium iodide showed higher AGE conversion (77.8%) and turnover (TON) (48.2%) than the other synthesized SILs under solvent-free conditions at 110 psi CO_2_ pressure. In addition, the researchers probed the effect of zinc halide doping of the SILs on the conversion and selectivity. Thus, the prepared IMIS-zn containing chloride anion was more active than the corresponding IMIS (without zinc) [38]. Silica-modified magnetic nanoparticles (MNPs) loaded with imidazolium ILs were also used for the cycloaddition of CO_2_ into styrene oxides at 1 MPa pressure [39].

Researchers prepared porous polymer-carrier PVIm loaded with alkyl chains of different alkyl chain lengths and halide anions for catalyzing the cycloaddition reaction of CO_2_ with AGE. Among the synthesized SILs, PVIm2-BuI(poly(n-vinylimidazole-codiinylbenzene) loaded with 1-iodobutane, the carrier with a pore size of 75.5 Å and a surface area of 952 m^2^ g^−1^, performed well and catalyzed the coupling reaction at a pressure of 1.34 MPa CO_2_, with high conversion of 92% and a TON of 77% [40]. The researchers carried out a 2.5 MPa CO_2_ pressure; chloromethylated polystyrene (CPS)-loaded hydroxyl-functionalized SILs (PS-HEIMX, X = I, Br, Cl) were prepared by the coupling reaction of CO_2_ with propylene oxide (PO), as shown in Figure 5c [37]. The easy-to-separate PS-HEIMBr was easier to separate compared to the other SILs synthesized by halide anion [41]. Due to the synergistic effect, the catalytic conversion and selectivity were highly improved [42].

### 2.5. Plasmonic Ionic Liquids

**Figure 6:**
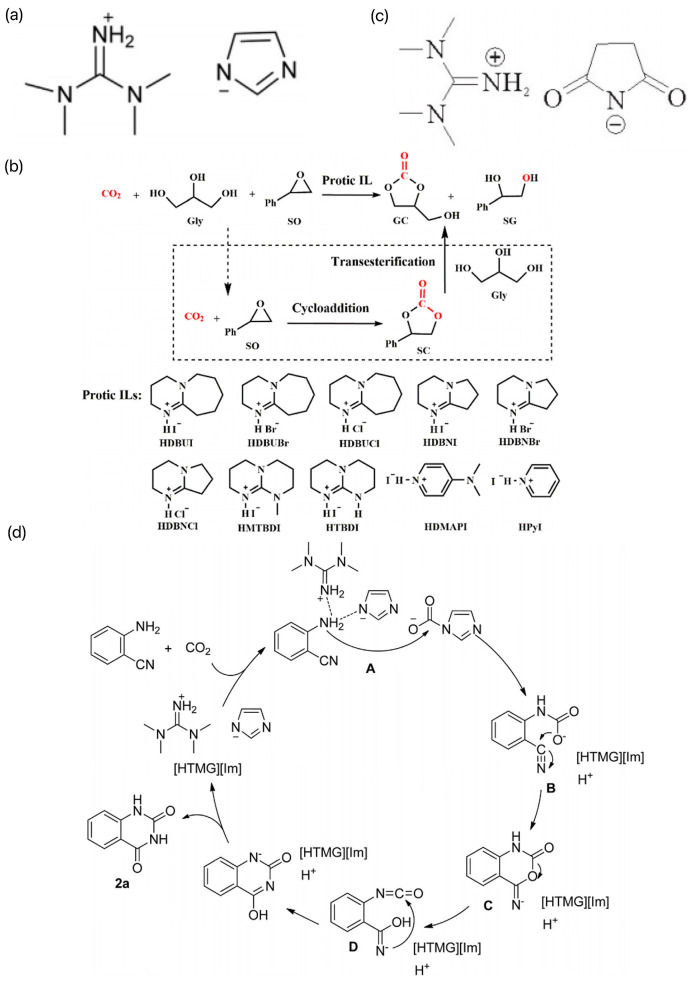
(**a**) Structure of [HTMG][Im] [43]. (**b**) Possible coupling reaction mechanism of CO_2_ with o-aminobenzonitrile [43]. (**c**) Structure of several multifunctional succinimide-based ILs [44]. (**d**) One-pot synthesis of high value-added GC and its byproducts Z-benzene glycol (SG) and styrene carbonate (SC) [45].

He et al. prepared a series of protonated ILs by a simple “one-step” neutralization reaction between a strong base and a weak protonated acid and investigated their applications as solvents and catalysts in the carboxylation reaction of CO_2_ with o-aminobenzonitrile [43]. Among them, the ILs [HTMG][Im] constructed by the neutralization reaction between TMG and imidazole had the best catalytic activity, and its structure is shown in Figure 6a. The coupling reaction of CO_2_ and o-aminobenzonitrile was catalyzed at room temperature and pressure without additional solvent by extending the reaction time appropriately, and the product yield could be up to 90%. A possible catalytic reaction mechanism was proposed, as shown in Figure 6b. On this basis, Liu et al. designed and synthesized several multifunctional succinimide-based ILs, the structures of which are shown in Figure 6c [44]. The ILs have the dual roles of solvent and catalyst, among which [HTMG][Suc] can reach 98% yield at 60 °C and 6 h. However, the molar amount of catalyst required is three times that of the substrate, and even if the reaction is carried out for 24 h at ambient temperature, 88% yield can be achieved. The synergistic activation of CO_2_ and 2-aminobenzonitrile by [HTMG][Suc] provides a reference for the reaction mechanism. In recent years, Luo et al. investigated a series of easy-to-prepare plasmonic ILs to synthesize high-value-added GCs (gas-chromatography standards) and the byproducts, benzene Z-ene glycol (SG) and styrene carbonate (SC), in a one-pot synthesis using CO_2_, Gly, and styrene oxide (SO) as the feedstock. The synthesis process is shown in Figure 6d [45]. SO was chosen as the substrate, and the GC yield could reach 94% at 45 °C under atmospheric pressure with 1,8-diazabicyclo[5.4.0]undec-7-iodoene (HDBUI) as the catalyst. The experimental results and density-functional theory (DFT) calculations indicated that the high catalytic activity of HDBUI was due to the strong nucleophilicity and substrate activation resulting from the synergistic interaction of the protonated HDBU^+^ cation and I^−^ anion.

### 2.6. Non-Protonic Ionic Liquids

Wang et al. reported a series of catalysts based on imidazole anions, among which [Ch][Im] showed the best catalytic effect [46]. This catalyst promoted the cycloaddition reaction of 2-aminobenzonitrile with CO_2_ at atmospheric pressure and 80 °C. In the literature, flue gas was used for the first time to synthesize quinazoline-2,4(1H,3H)-dione, which was reacted at 50 °C and 0.15 bar CO_2_ pressure for 36 h, and still yielded a 93% isolated yield of the product, showing excellent catalytic activity. In addition, Wang et al. reported in 2019 a choline-based ILs catalyst that can catalyze the cyclization reaction of 2-aminobenzonitrile with CO_2_ at 40 °C under atmospheric pressure and obtained a 99% reaction yield [47]. Compared with the previously reported quaternary ammonium salt catalysts, the reaction conditions were milder. Meanwhile, the quaternary ILs have lower viscosity and higher decomposition temperature compared to the quaternary ILs catalyst. The quaternary salt [Bu,P]-2-MIm based on 2-methylimidazole anion reported by Liu et al. also gave excellent product yields with a reduced catalyst dosage and further improved catalytic efficiency at 80 °C and 1 bar CO_2_ pressure [48]. In this work, it was found that the synthesized ILs can activate atmospheric CO_2_ to form carbamates, which in turn, react with 2-aminobenzonitrile to synthesize quinazoline-2,4(1H,3H)-diones.

## 3. Application of Ionic Liquids in Catalytic CO_2_ Conversion

ILs have shown unique advantages in catalyzing and promoting CO_2_ conversion. In the process of CO_2_ conversion, ILs can be used as solvents, electrolytes, CO_2_ adsorbents, activators, catalysts, and co-catalysts to realize the chemical conversion of CO_2_ under mild and metal-free conditions and can be coupled with metal catalysts to realize their directed conversion and obtain high-value-added chemicals [49]. Table 1 shows the high catalytic activity and selectivity of ILs to the products. In the IL catalytic system, multiple roles coexist to synergistically realize the activation and conversion of CO_2_ [50]. The following discussion is based on the promotion of ILs in four aspects, namely photocatalytic conversion, electrocatalytic conversion, photoelectrocatalytic conversion, and photothermal catalytic conversion of CO_2_.

### 3.1. Photocatalytic Conversion of CO_2_ by Ionic Liquids

Most ILs exhibit good photostability under sunlight and utilize stable radicals or anions to activate CO_2_ [58]. Theoretical studies have shown that ILs may form excess charge localization or cavities under electrochemical or radiation effects, which makes them attractive materials for CO_2_ photoreduction [59,60]. In particular, imidazolium-based ILs are highly solar stable, and their mechanism for promoting CO_2_ activation is through the in situ formation of imidazolium CO_2_^−^ adducts, which are considered to be a key intermediate that can dissociate into BMIm^+^ and CO_2_^−^ radicals. Photocatalytic technology has attracted much attention due to its advantages of easy operation, low energy consumption, and no secondary pollution, but it still faces the challenges of easy compounding of electrons and holes, limited absorption of light by photocatalysts, and high activation energy of the reaction in practical applications. To overcome these problems, in addition to novel photocatalytic technologies that incorporate external fields, the photocatalytic conversion efficiency of CO_2_ can be improved by compounding photocatalysts with other compounds, such as ILs [61,62,63].

**Figure 7:**
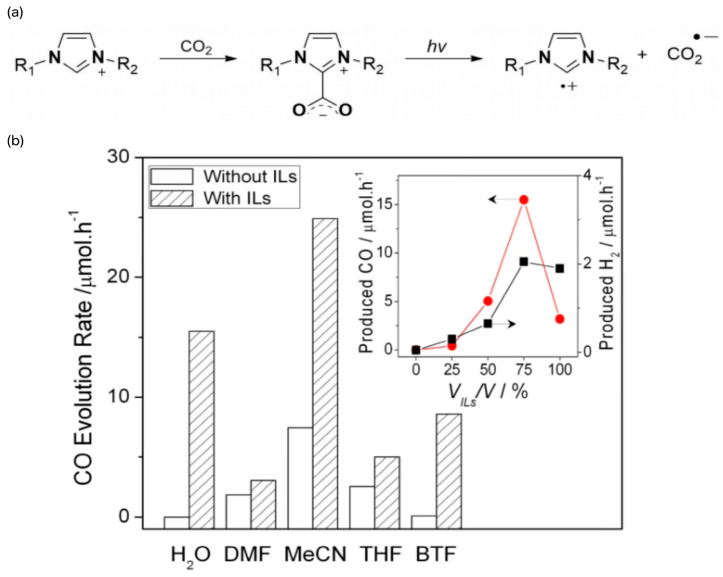
(**a**) Formation of imidazoliumCO_2_-adduct and its radical intermediates generated under irradiation [49]. (**b**) Promotion of CO_2_ photocatalysis by different ILs. The production of CO and H_2_ increases with the increase of the [EMIM][BF_4_]/H_2_O ratio. However, when H_2_O was removed, the activity of CO and H_2_ formation slowed down significantly [52].

For example, imidazolium-based ILs can be used as photocatalysts to realize the reduction of CO_2_ in water without the need for semiconductors or scavengers. As an example, the amphoteric IL 1-n-butyl-3-methylimidazole-2-carboxylate (BMIm-CO_2_) has an apparent quantum yield of CO as high as 250 μmol/L and an apparent quantum yield of 3.9%. The reaction mechanism involves the generation of BMIm radical cations ([BMIm]^+^) and CO_2_ radical anions (CO_2_^−^) via the homolytic cleavage of CO_2_, a process that is validated both experimentally and theoretically [49]. The reaction mechanism is shown in Figure 7a. Peng et al. studied the relationship between the CO_2_ photoreduction efficiency, UV/Vis adsorption capacity, and Kamlet–Taft (K-T) β-parameter value of BMIm· Oac ILs in water and isopropyl alcohol [64]. We believe that the activation of CO_2_ is related to the properties of anions, which drive the formation of CO_2_^−^ reaction intermediates. It was also observed that the pH value of ILs significantly promoted CO_2_ photoreduction. For example, BMIm·OC_6_F_5_ ILs at pH 7.18 strongly induce CO_2_ reduction by increasing CO_2_ solubility, producing large amounts of HCO_3_^−^, which is subsequently reduced to CO and CH_4_. Wang’s group found that, under mild conditions (1 atm CO_2_ and visible light), ILs can promote the photoconversion of CO_2_ to CO using [Ru(bpy)_3_]Cl_2_ as a photocatalyst [52]. The relevant test results are shown in Figure 7b. The high promotive properties of ILs are due to the task specificity of ILs and the functional groups at the ILs site.

On the other hand, Chen et al. demonstrated a CO_2_ uptake capacity of up to 3.26 mol CO_2_/mol ILs at room temperature by designing novel ILs such as tetra-coordinated ILs obtained by the neutralization reaction of citric acid with tetrabutylphosphine hydroxide [51]. These ILs complexed with CO_2_ (IL-CO_2_) exhibited visible-light absorption properties and promoted the photocatalytic reduction of CO_2_ to CH_4_ by anatase-type TiO_2_ under light conditions, with yields up to 3.52 μmol/g per hour and selectivities up to 96%. Mechanistic studies showed that IL-CO_2_ complexes acted as photosensitizers to promote the photocatalytic reduction of the CO_2_ reaction under visible-light irradiation.

These studies show the potential and advantages of ILs in a variety of photocatalytic conversion reactions and provide new ideas and possibilities for the development of efficient and environmentally friendly CO_2_ conversion technologies.

### 3.2. Electrocatalytic Conversion of CO_2_ by Ionic Liquids

In recent years, the application of ILs as a reaction medium in the electrochemical reduction of CO_2_ has shown remarkable catalytic activity and unique advantages. Its main mechanism of action can be summarized as follows. First, compared with the traditional aqueous solution system, CO_2_ has a higher solubility in ILs, which is conducive to improving the mass transfer rate in the electrochemical reduction process of CO_2_, thus enhancing the driving force of the CO_2_ reaction and the conversion rate of products. Second, ILs can effectively inhibit the hydrogen evolution reaction in water, thus improving the selectivity of the product. In addition, ILs provide a superior microenvironment at room temperature, which can activate CO_2_ molecules more effectively and promote the reduction reaction of CO_2_ [65,66,67]. Therefore, the highly efficient activation and catalysis of ILs in the field of CO_2_ electrochemical reduction has gradually become the forefront of research.

**Figure 8:**
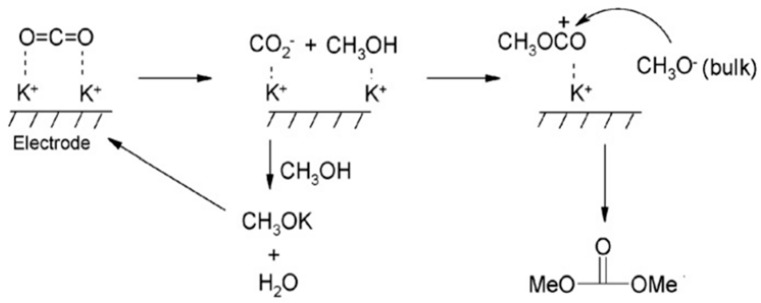
Possible mechanisms for the preparation of dimethyl carbonate [68].

Researchers have made remarkable progress in the electrochemical reduction reaction of CO_2_, especially when using ILs as the reaction medium. These studies mainly focus on improving the efficiency and selectivity of the reaction. For example, Rosen et al. have made significant progress in the conversion of CO_2_ to CO through the method of direct electrocatalytic reduction [65]. By introducing ILs, the overpotential of CO_2_ reduction is successfully reduced, resulting in highly selective CO generation. The researchers further found that, by adding water to [EMIM]BF_4_ and using Ag as the catalytic electrode, not only can the CO_2_ reduction overpotential be reduced but also the CO_2_ conversion frequency can be increased. Cai et al. used Pt as the working electrode to prepare dimethyl carbonate by the electrocatalytic reduction of CO_2_ in 1-butyl-3-methylimidazole bromide (BMIMBr)-potassium methanol-methanol medium using potassium methanol as a cocatalyst [68]. The reaction mechanism is shown in Figure 8. Although this method can simplify the separation process of the product, the yield of the system is only 3.9%. When substituting propylene oxide for potassium methanol, the yield can be increased to more than 75.5%. In addition to the electrochemical conversion of CO_2_ to CO and HCOOH, another highly anticipated application is the conversion of CO_2_ to methanol, which has been extensively studied in ILs. Han and his team converted CO_2_ to methanol by electrochemical activation [69]. The team used a variety of bimetallic catalysts, such as Mo-Bi, Mo-Ag, and Mo-Cu, in the [BmIm][BF_4_]/acetonitrile solution. In 0.5 M IL-acetonitrile solution, Mo and Bi metals work together as bimetallic catalysts to achieve 71.2% Faraday efficiency for the electrochemical activation of CO_2_. In bimetallic MO-Bi catalysts, Mo promotes the conversion of CO_2_ to CO, while Bi contributes to H_2_ generation and in situ adsorption of CO, thus increasing the possibility of CO_2_ being hydrogenated to CH_3_OH.

### 3.3. Photoelectrocatalytic Conversion of CO_2_ by Ionic Liquids

Photoelectrocatalysis uses solar and electrical energy to catalyze the conversion of CO_2_ into organic matter. Compared with photocatalysis, appropriately applied bias can cause energy-band bending, which contributes to the orderly transfer of photogenerated electrons and thus reduces the recombination of photogenerated electron–hole pairs [70]. In addition, even if the energy-band position of the photocatalyst is unfavorable for CO_2_ reduction or H_2_O oxidation, it can still be used for the photoelectrocatalytic reduction of CO_2_ under appropriate applied bias conditions [71].

Lu et al. introduced an IL (1-aminopropyl-3-methylimidazolium bromide salt) to enhance the reaction system for the photoelectrocatalytic reduction of CO_2_ in order to increase the solubility of CO_2_ in water and facilitate its catalytic conversion [72]. The solution acted as both absorber and electrolyte at ambient temperature and pressure. Under an applied voltage of 1.7 V, the Faraday efficiency of formic acid was up to 94.1%, which was significantly higher than the photoelectrocatalytic systems without the introduction of ILs (37.2%) and with the introduction of ILs without an amino group (62.4%). In this photoelectrocatalytic reduction system, the amino-containing ILs critically facilitated the conversion of CO_2_ to formic acid and inhibited the generation of H_2_. Cronin’s team performed a CO_2_ to CO photoelectric conversion study in a non-aqueous [EMIM][BF_4_]/CH_3_CN solution, obtaining a Faraday efficiency of 99% at an underpotential of +0.78 V [73]. The photocatalytic conversion of CO_2_ to CO was investigated by Cronin’s team in a non-aqueous [EMIM][BF_4_]/CH_3_CN solution. It was shown that the photocatalytic yield increased with the increase in the number of TiO_2_ layers, which is believed to be due to the increase in the catalytically active sites and thus the rate of electron–hole pair complexation. In addition, the formation of complexes between [EMIM]^+^ ions and CO_2_ lowered the energy barrier of the reaction, which further contributed to the efficiency of the photoelectric conversion.

**Figure 9:**
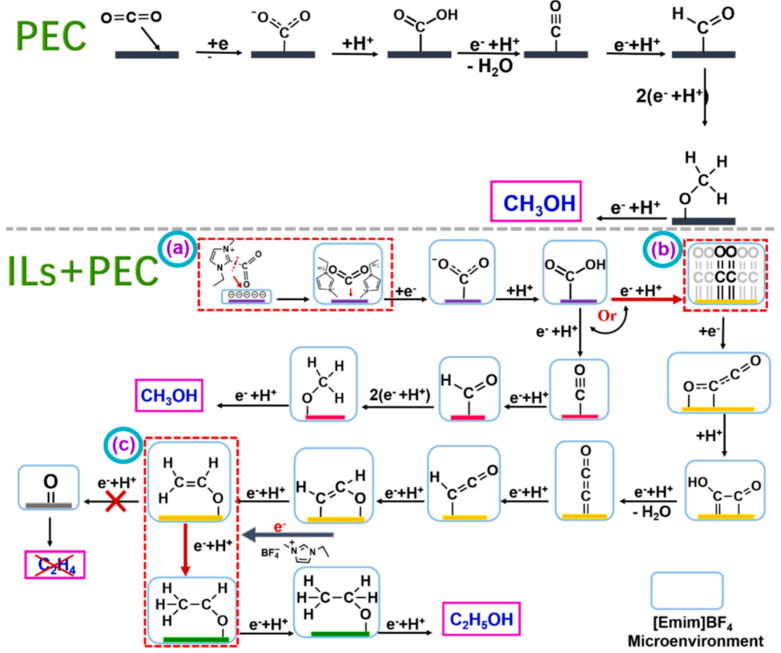
The mechanism of photoelectrocatalytic CO_2_ reduction over Cu_2_O/TiO_2_ catalyst in the presence of [Emim]BF_4_. (Step a) CO_2_ is complexed with [Emim*] to form [Emim*−CO_2_] complex. (Step b) Two adjacent *C=O intermediates form *C_2_O_2_^−^ through electron transport−mediated facilitation, which in turn produces C2. However, a small percentage of *C≡O intermediates are converted to CH_3_OH. The coupled intermediate *C_2_O_2_^−^ is converted to the intermediate CH_2_CHO* by a series of proton and electron transfer processes. This reaction produces either ethylene or ethanol. (Step c) The resulting CH_3_CHO* intermediate undergoes electron and proton transfer to produce CH_3_CH_2_OH [55].

Gao et al. prepared Cu_2_O/TiO_2_ nanoarray electrode materials using anodic oxidation and electrodeposition methods and applied them to the photocatalytic reduction of CO_2_ [55]. The mechanism of photoelectrocatalytic CO_2_ reduction over a Cu_2_O/TiO_2_ catalyst in the presence of [Emim]BF_4_ is shown in Figure 9. In the system without [Emim]BF_4_, CO_2_ molecules approach and bind to the active site on the catalyst surface to form the intermediate CO_2_^−^. This process requires overcoming a large activation energy. This intermediate is then protonated to produce HOCO, which is then converted into the key intermediate *CO through a series of electron acquisition, proton acquisition, and dehydration reactions. *CO intermediates can exist in two forms: C≡O and C=O. The former is important for the formation of C1 products, while the latter is important for the formation of C–C coupling to produce multi-carbon products. Without the addition of [Emim]BF_4_, the intermediate product *C≡O can be eventually converted into products such as methanol through a series of proton and electron transfer processes [74,75,76]. However, once [Emim]BF_4_ is added, the reaction path of the system changes significantly. [Emim]BF_4_ has a strong ability to capture CO_2_ and enrich CO_2_ on the catalyst surface, thus creating a high-concentration CO_2_ microenvironment on the electrolyte and catalyst surface. In addition, imidazole cation [Emim]+ forms [EMIM-CO_2_] complexes with CO_2_ in the catalyst–electrolyte interface region, further affecting the kinetics and reaction path of the reaction. This complex will improve the CO_2_ adsorption and conversion efficiency of the catalyst surface, thereby improving the selectivity and reaction rate of the final product [77]. It was found that the addition of [Emim]BF_4_ significantly improved the yield of the alcohol product, resulting in 82.7% selectivity for ethanol. The researchers delved into the promotion mechanism of [Emim]BF_4_ and concluded that the enrichment of [Emim]BF_4_ in the cathode region enhanced the capture of CO_2_. In addition, [Emim]BF_4_ formed a strong interaction with the electrode surface, which effectively improved the separation efficiency and migration rate of photogenerated charges.

### 3.4. Photothermal Catalytic Conversion of CO_2_ by Ionic Liquids

The photothermal synergistic catalytic technology combining solar and thermal energy utilizes the integrated photothermal effect to enhance the performance of the catalyst. Meanwhile, the thermal effect had a significant impact on the CO_2_ absorption by ILs [78].

Bai et al. tested the thermal stability of four 1-alkyl-3-methylimidazolium bis(trifluoromethylsulfonyl)imides ([C_n_MIm][Tf_2_N]) for CO_2_ absorption at 393.15 K [79]. The results showed that [C_n_MIm][Tf_2_N] was the most effective catalyst for CO_2_ absorption. The results showed that [C_n_MIm][Tf_2_N] exhibited long-term thermal stability at 393.15 K. The results showed that [C_n_MIm][Tf_2_N] was a good choice for the thermal stability of CO_2_ uptake. The solubility of CO_2_ in [C_n_MIm][Tf_2_N] increased significantly with increasing pressure in the range of 353.15 K to 393.15 K and decreased slightly with increasing temperature. In addition, the solubility of CO_2_ in the ILs increased with the increase of alkyl chain length on the cation.

**Figure 10:**
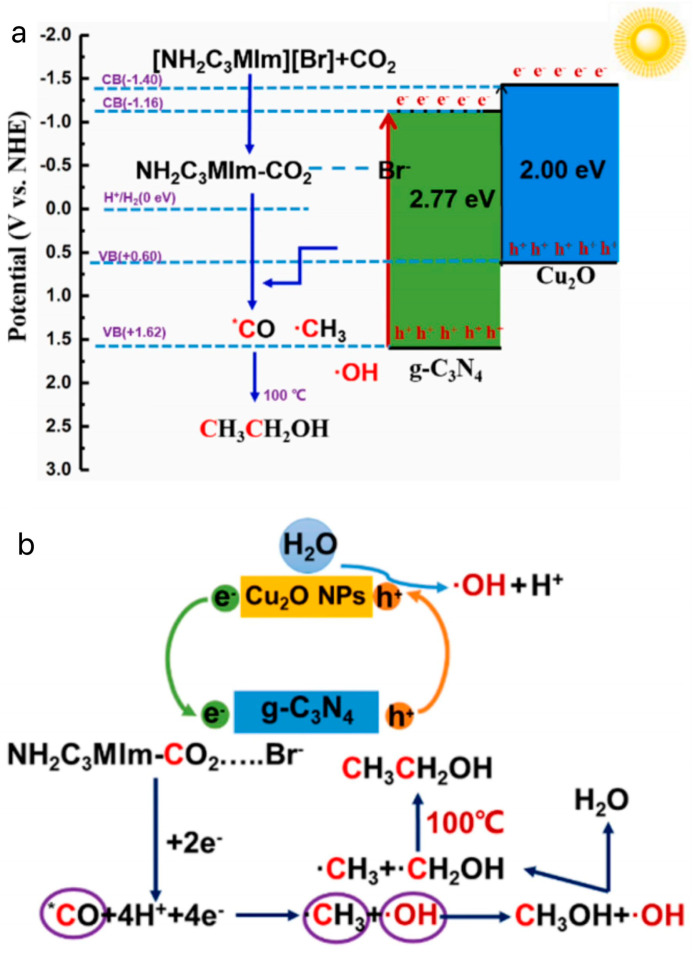
(**a**) Principle of photothermal catalyzed conversion of CO_2_ to CH_3_CH_2_OH [80]. (**b**) The possible reaction pathways of Cu_2_O/g-C_3_N_4_ in [APMIm][Br] aqueous solution [80].

Li et al. synthesized Cu_2_O/g-C_3_N_4_ heterojunctions by a simple hydrothermal method to photothermally catalyze the conversion of CO_2_ to CH_3_CH_2_OH in an aqueous solution with the addition of the bromine salt of 1-aminopropyl-3-methylimidazole [80]. The photothermal catalytic conversion of CO_2_ to CH_3_CH_2_OH is shown in Figure 10a. Figure 10b shows the possible reaction pathways of Cu_2_O/g-C_3_N_4_ in an [APMIm][Br] aqueous solution. First, Cu_2_O and g-C_3_N_4_ are excited by light to generate e^−^ and h^+^ ions. The transfer of e— from the CB in Cu_2_O to the CB in g-C_3_N_4_ promotes rapid separation of the carrier. Second, the CO_2_ adsorbed by Cu_2_O continuously complexes with [AMPIm][Br] to form APMIm-CO_2_, which can reduce the overpotential and react with g-C_3_N_4_ to form *CO. It has been reported that ILs provide a low-energy pathway by forming C_2_MIm-CO_2_ complexes, which can easily convert CO_2_ into *CO and promote continuous coupling of C–C under visible-light irradiation, which is an important reason for improving the ethanol yield [81,82,83]. In addition, continuous proton and electron transfer completed the conversion from *CO to ^•^CH_3_. In addition, the thermal field can accelerate the motion and collision between ^•^OH and ^•^CH_3_, further promoting C–C coupling to generate ethanol. Finally, a series of free-radical reactions and proton–electron transfer processes are completed to achieve the conversion of CO_2_ to ethanol. Specifically, the consumption of *CO can reduce the coverage of *CO on the surface of Cu_2_O/g-C_3_N_4_, promote the reduction of CO_2_ to CH_3_CH_2_OH, and weaken the toxic effects of *CO. The yield of CH_3_CH_2_OH reached 0.71 mmol·g^−1^·h^−1^, which is higher than that of the pure aqueous reaction system (0.56 mmol·g^−1^·h^−1^), which was 1.27 times higher than that of the pure water reaction system (0.56 mmol·g^−1^·h^−1^). Due to the presence of amino groups in the ILs, the solubility of CO_2_ in the reaction system was effectively increased. In addition, the current density of the ILs electrolyte solution was 21.3 mA·cm^−2^, which was higher than that of the KHCO_3_ electrolyte solution of 12.8 mA·cm^−2^, and lowered the overpotential of 0.34 V, which promoted the reduction of CO_2_ by reducing the polarization. The ILs played a key role in inhibiting H_2_ generation.

## 4. Valuable Chemicals

### 4.1. CO_2_ Hydrogenation

The hydrogenation of CO_2_ to produce chemical products and fuels is a high-efficiency and energy-saving method that is of wide interest to the chemical industry. By changing the valence of the carbon atoms, CO_2_ can be reduced to different products, in the order of HCOOH, CH_2_O, CH_3_OH, and CH_4_. In particular, long-chain hydrocarbons, such as C2–C4, will be the focus of future research [84].

Han et al. developed a reaction–separation system to synthesize formic acid from CO_2_ (4–18 MPa) and H_2_ (1–9 MPa) under the catalysis of amino ILs using ruthenium immobilized on silica as a heterogeneous catalyst at 60 °C [85]. Subsequent product recovery and catalyst regeneration can be easily achieved through filtration and evaporation. The key was to design ILs with a specific function, with a tertiary amine group on the cation, capable of forming a salt with formic acid. Although some progress has been made by designing diamine-functionalized ILs, the reaction conditions remain challenging. The reaction temperature was raised to 150 °C while maintaining corresponding pressure conditions (hydrogen gas 4–6 MPa, total H_2_/CO_2_ 8 MPa) [86]. Under these conditions, the optimum CH_4_ yield is 69%. Further increasing the reaction temperature or increasing the amount of catalyst can improve the methane yield and TON. In addition, the study showed that the increase rate of TON was more significant in the temperature range from 140 °C to 150 °C, while the product formation was not observed below 120 °C.

### 4.2. C–O Bond

Typical reactions to build C–O bonds typically require highly active reagents, such as organolithium reagents or Grignard reagents. The substrates of these reactions may include alkenes, dienes, alkynes, halogenated aromatic compounds, epoxides, and aromatic compounds containing hydrocarbon groups. However, the use conditions of organolife and Grignard reagents are very strict, which limits their widespread use in practical applications. ILs, as a green and sustainable solvent, offer new opportunities in the process of building C–O bonds, especially in reactions with carbon dioxide and other substances. However, to date, ILs-mediated C–O bond formation reaction products have been largely limited to the form of carbonates, including cyclic and linear carbonates.

Wang’s research group conducted an interesting study using epoxides instead of propargyl alcohol for the cycloaddition reaction of CO_2_ [87]. At a CO_2_ pressure of 1 bar and a temperature of 60 °C, α-alkylene cyclocarbonate, using 200 mol% ILs with a yield of up to 89% and without the use of other solvents, was successfully synthesized. This study shows that ILs can be used not only as solvents or reactants but also as effective catalysts or reaction templates under mild conditions [88]. In some systems, eutectic ILs show excellent selectivity and efficiency in the cycloaddition reaction of CO_2_ and epoxide, which provides a new way and possibility for green chemical synthesis [89]. The study by Zhao et al. demonstrates a new method for the efficient synthesis of linear dimethyl carbonate using bicarbonate ILs as a recyclable catalyst and dehydrating agent [90]. The team took advantage of the high tunability of IL bicarbonate and the high polarity, high solubility, and easy activation of CO_2_. In this study, the reaction conditions were relatively mild, using 1 MPa of CO_2_ and methanol, and carried out at near room temperature. This method not only avoids the toxic substances used in traditional synthesis methods but also utilizes the properties of ILs to achieve a highly efficient synthesis process. This innovative approach provides an important advance for the sustainable preparation of carbonate compounds and promotes the development of green chemical synthesis.

### 4.3. C–N Bond

The formation of a carbon–nitrogen bond is important for the synthesis of organic substances and intermediates. In recent years, the use of ILs to construct C–N bonds from CO_2_ has become a promising approach. ILs can be used as a CO_2_ adsorbent, activator, reaction catalyst, substrate solvent, and product separator. In addition, its high adjustability, high stability, and low volatility make it a recyclable solvent and catalyst, conducive to the concept of green chemistry and sustainable development.

Choi et al. demonstrated a method of catalyzing the formation of carbamate from amines, CO_2_, and silicate esters using superbasic protonated ILs [DBU][Ac] at 5 MPa CO_2_ and 150 °C acetonitrile [91]. The team observed that the activity of aromatic amines was lower than that of fatty amines due to their lower pKa values, which is related to hydrogen bond interactions between basic acetic acid anions in ILs. Despite the success of this study, the disadvantages include the use of a cosolvent (acetonitrile) and the relatively strict reaction conditions (5 MPa CO_2_ and 150 °C). In order to improve this method, future studies can try to design new types of metal-ion-free liquids to achieve a more moderate temperature and lower CO_2_ pressure that still can efficiently catalyze the synthesis of CO_2_ carbamate. This improvement is expected to improve the sustainability and practicality of the reaction and promote the further development of the application of ILs in CO_2_ conversion chemistry. He et al. designed a series of Lewis bases and cationic ILs to catalyze the synthesis of chemically and regionally selective 5-aryl-2-oxazolidinones from azacil and CO_2_ without the need for any organic solvents or additives [92,93]. Unfortunately, these conditions are relatively stringent, requiring, for example, a CO_2_ pressure of 3–9 mpa. 2-oxazolidinone using copper-substituted polyoxometalate-based ILs at one atmosphere of CO_2_ and 25 °C was successfully synthesized by improving the carboxylated cyclization of various propylamines through the dual activation of propylamine and CO_2_. However, the disadvantages of using metals such as copper provide room for improvement in the synthesis of oxazolidinone. Han et al. synthesized 3,4, 5-trisubstituent oxazolone using propylamine and CO_2_ at 100 °C and one atmosphere of CO_2_ [94]. Relevant researchers also improved the conditions and catalyzed the proton-type ILs reaction of propylamine to 2-oxazolidinone at 1 atmosphere CO_2_ and 60 °C [95].

### 4.4. C–S Bond

Liu et al. reported for the first time the efficient synthesis of C–S bonds in benzothiazole using CO_2_, 2-aminothiophene, and hydrosilane catalyzed by a metal-ion-free liquid at 40 °C and 5 MPa CO_2_ [96]. The team found that [BMIM][Ac] exhibited the best catalytic activity and was able to activate CO_2_, hydrosilane, and 2-aminothiophene through hydrogen bonding. The reaction mechanism is as follows: (1) formation of intermediates: first, intermediate A and hydrosilane/A complex B are formed; (2) attack: subsequently, methoxysilane C attacks 2-aminothiophene, forming the intermediate D; (3) release: hydrosilane and water are released through other intermediates E and F, resulting in the final product G. The main contribution of this study is to summarize the mechanism of C–N bond and C–S bond construction by CO_2_ in ILs media. To date, this is the only study that uses ILs as a catalyst to construct C–S bonds from CO_2_. Under the conditions of low CO_2_ pressure and no hydrosilane, ILs as a catalyst and a solvent can maintain high selectivity and high efficiency, which provides valuable consideration for future research.

### 4.5. C1 Products

Brennecke et al. found that the use of [EMIM][Tf_2_N] as the electrolyte promoted the formation of CO on the Pb electrode, but not the formation of oxalate anion [83]. With the increase of [EMIM][Tf_2_N] concentration, the tendency of CO formation also increased, and carboxylate was formed.

Han et al. used supercritical CO_2_ as a carbon source for CO_2_ reduction on the Cu cathode and found that the main product was CO in the ionic liquid medium [BMIM][PF_6_] [97]. The team has successfully demonstrated that both the product and the ILs can be effectively recycled without cross-contamination problems. In addition, Rosenzweig’s group also explored modifying the product selectivity of CO_2_ reduction reactions by changing ILs [98]. Under the same bismuth cathode conditions, the reduction of CO_2_ to HCOO^−^ or CO products can be controlled by choosing different ILs. Han’s research group further developed a metal-free catalyst (graphene oxide–multi-walled carbon nanotube composite) that exhibited higher electrical activity than conventional Au and Ag electrodes in ILs/CH_3_CN media, particularly in the reaction of reducing CO_2_ to CO [99]. These studies show that ILs have great potential for application in the process of CO_2_ electroreduction, and more efficient CO_2_ conversion can be achieved by adjusting the properties of ILs and selecting suitable catalysts.

The study by Han et al. has pioneered a new pathway for the efficient and highly selective electrochemical conversion of CO_2_ to CH_4_ by combining a metal–organic framework (MOF) cathode and a pure ILs electrolyte [100]. Their study shows that this approach utilizes intermediates between CO_2_ and CO for a more efficient CO_2_ reduction process. Regarding the choice of ILs, it was shown that fluorine-containing imidazolium-based ILs (e.g., [BMIM][PF_6_] > [BMIM][BF_4_] > [BMIM][TFO]) were more efficient than non-fluorine-containing ILs (e.g., [BMIM][ClO_4_]) for the CO_2_ electroreduction reaction. This is due to the stronger interaction between fluorinated ILs and CO_2_, which enhances the solubility and activation of CO_2_ and, thus, promotes efficient electrochemical conversion. In addition, Jovanovic’s group used [BMIM][BF_4_] as a medium in their experiments based on a microscale electrochemical reactor to develop a mathematical model of the CO_2_ reduction reaction. This model not only describes the processes involving CO_2_ and H_2_O as reactants but also predicts the generation of products including CH_4_, CH_3_OH, H_2_, HCOOH, and HCHO [101]. This mathematical model helps to understand and optimize the conditions of the CO_2_ electroreduction reaction to achieve higher product selectivity and efficiency. In summary, the work of Han et al. provides a new direction for the development of sustainable energy and chemical production by utilizing a combination of MOFs and fluorinated ILs to achieve efficient conversion of CO_2_, which is important for mitigating climate change and enhancing energy efficiency.

### 4.6. C–C Bond

To our knowledge, there have been no reports about the C–C bond construction of CO_2_ using thermal energy in ILs. However, with the help of electrochemical action, the construction of C–C bonds by CO_2_ in ILs can be achieved with relative ease. Atobe and his team found that CO_2_ solubility in [DEME][TFSI] increased significantly under supercritical CO_2_ conditions. An ILs/supercritical CO_2_ system facilitates the carboxylation of various organic halides on Pt, Cu, GC, and Ag electrodes.

In addition, by increasing the pressure of CO_2_ and using the precious metal Pt [102], Dai et al. devised an innovative, direct, and efficient method for reacting CO_2_ at atmospheric pressure with benzyl bromide to produce ethyl phenylacetate in [BMIM][BF_4_], which is the main product. A Cu-Ni electrode is considered to be the key reason for the efficient reaction because of its high porosity and good adsorption capacity of CO_2_ and substrate [103]. Huang et al. used a [BMIM][BF_4_] electrolyte to construct a C–C bond between CO_2_ and aromatic ketone and synthesized α-hydroxy-carboxylic acid methyl ester [104]. In addition, the results show that temperature, current density, total charge, electrode, and reactant concentration have significant effects on the yield of methyl α-hydroxy-carboxylate. Hiejima’s team found that, by increasing the pressure and temperature of CO_2_ on the platinum electrode, they could significantly improve the electrochemical carboxylation efficiency of alpha-chloro-ethylbenzene and successfully construct the C–C bond [105]. Huang’s team avoided the use of volatile/toxic solvents, catalysts, or other auxiliary electrolytes, and successfully carboxylated 2-amino-5-bromopyridine with CO_2_ using only ILs as the medium, with a yield of 75% and a selectivity of 100% for the product 6-amino-niacin [106].

### 4.7. C–N Bond

The Inesi team first obtained the electrically generated O_2_^•−^ substance by electroactivating O_2_/CO_2_ in ILs. Then, using these electrically generated O_2_^•−^ substances and amines and CO_2_, the team successfully constructed C–N bonds to synthesize carbamates, of which 2-imidazolone is a byproduct. This process requires a lower negative potential than the direct CO_2_ electroreduction method and avoids the use of other volatile and toxic organic solvents, auxiliary electrolytes, and catalysts [107]. Studies have also shown that Ni cathodes exhibit the best electrocatalytic activity at various types of electrodes, such as C, Cu, Pt, Pb, and Cu. In addition, in a [BMIM][BF_4_] solution containing amines, CO_2,_ and the alkylating agent EtI, organic carbamates were successfully synthesized using amines and CO_2_. In this process, without the use of other solvents and catalysts, the C–N bond is formed at 1.0 atm CO_2_ and 55 °C. The study also shows that Pt electrodes exhibit the most effective cathode properties in Ni, Cu, and Pt [108].

## 5. Conclusions

ILs, with their unique structure and properties, are widely used in many fields, including as CO_2_ adsorbents, activation promoters, electrolytes/solvents, catalysts, or cocatalysts. We discussed different types of ionic liquid catalysts and their catalytic mechanisms. ILs play an important role in the photocatalytic conversion, electrocatalytic conversion, photocatalytic conversion, and photothermal catalytic conversion of CO_2_. The type of catalyst, operating temperature, pressure, and other factors (such as solvents or cocatalysts) all have an impact on the efficiency and selectivity of CO_2_ conversion. By optimizing the solubility of CO_2_ in the reaction system, reducing the overpotential of the electrocatalytic reaction, and reducing the activation energy of the reaction, the efficient catalytic conversion of CO_2_ was realized. ILs, as controllable “green solvents”, show their importance in various applications. Although remarkable progress has been made in the field of catalytic conversion of CO_2_ into high-value-added products, several challenges remain.

(1) Although functional ILs have multiple functions in CO_2_ conversion, problems such as high price, poor stability, and high viscosity still need to be solved. Because ILs are highly designable, they can be modified to meet a variety of requirements for industrial applications, such as reducing cost, improving stability, reducing viscosity, enhancing efficiency, increasing selectivity, and facilitating separation;
(2) Combining metal-free photocatalysts with task-specific ILs to capture CO_2_ from the air and simulate artificial photosynthesis to produce high value-added products is a potential research direction in the future;
(3) Consider the use of low-cost metal-free catalysts for thermal conversion of CO_2_ at room temperature and pressure to improve economy and practicality;
(4) ILs should be stable during thermal, electrical, and photocatalytic processes. Many ILs may break down or react with other chemicals after prolonged exposure to the environment. In particular, the problem to be solved is how to improve the stability of functionalized task-specific ILs.

## Figures and Tables

**Table 1:** High selectivity of ionic liquids to products.

ILs	Catalytic Mode	Catalytic Condition	Catalyst	Sel%	Ref.
[P_4444_]_3_[p-2,6-O-4-COO]	Photocatalytic conversion	visible light > 420 nm	CH_4_	96.2%	[51]
[Ru(bpy)_3_]Cl_2_	Photocatalytic conversion	1 atm, visible light > 420 nm	CO	96.3%	[52]
ApmimBr	Electrocatalytic conversion	20 °C, voltage 3.5 V,	C_3_H_6_O_3_	94.5%	[53]
[APMIm]DCA	Electrocatalytic conversion	50 °C	4-(hydroxymethyl)-1,3-dioxolan-2-one	96.8%	[54]
[Emim]BF_4_	Photoelectrocatalytic conversion	visible light, −0.9 vs. NHE	CH_3_CH_2_OH	82.7%	[55]
[EmimOH]/[NTF_2_]/DBU/SmOCl	Thermocatalytic conversion	140 °C	C_3_H_6_O_3_	99.1%	[56]
BzMDH	Thermocatalytic conversion	140 °C	C_3_H_6_O_3_	99.7%	[57]

## Data Availability

Not applicable.
